# The Impact of Monetary Incentives on Physician Prosocial Behavior in Online Medical Consulting Platforms: Evidence From China

**DOI:** 10.2196/14685

**Published:** 2019-07-26

**Authors:** Dong Jing, Yu Jin, Jianwei Liu

**Affiliations:** 1 Key Laboratory of Interactive Media Design and Equipment Service Innovation Harbin Institute of Technology Harbin China; 2 School of Management Harbin Institute of Technology Harbin China

**Keywords:** online medical consultation, monetary incentives, physicians’ prosocial behaviors, self-recognition, others’ recognition

## Abstract

**Background:**

In online medical consulting platforms, physicians can get both economic and social returns by offering online medical services, such as answering questions or sharing health care knowledge with patients. Physicians’ online prosocial behavior could bring many benefits to the health care industry. Monetary incentives could encourage physicians to engage more in online medical communities. However, little research has studied the impact of monetary incentives on physician prosocial behavior and the heterogeneity of this effect.

**Objective:**

This study aims to explore the effects of monetary incentives on physician prosocial behavior and investigate the moderation effects of self-recognition and recognition from others of physician competence.

**Methods:**

This study was a fixed-effect specification-regression model based on a difference-in-differences design with robust standard errors clustered at the physician level using monthly panel data. It included 26,543 physicians in 3851 hospitals over 133 months (November 2006-December 2017) from a leading online health care platform in China. We used the pricing strategy of physicians and satisfaction levels to measure their own and patients’ degree of recognition, respectively. Physicians’ prosocial behavior was measured by free services offered.

**Results:**

The introduction of monetary incentives had a positive effect on physician prosocial behavior (β=1.057, *P*<.01). Higher self-recognition and others’ recognition level of physician competence increased this promotion effect (γ=0.275, *P*<.01 and γ=0.325, *P*<.01).

**Conclusions:**

This study explored the positive effect of the introduction of monetary incentives on physician prosocial behavior. We found this effect was enhanced for physicians with a high level of self-recognition and others’ recognition of their competence. We provide evidence of the effect of monetary incentives on physicians’ prosocial behaviors in the telemedicine markets and insight for relevant stakeholders into how to design an effective incentive mechanism to improve physicians’ prosocial engagements.

## Introduction

Online medical service platforms are growing rapidly. They have been viewed as an important supplement for the offline health care industry through medical resource allocation and physician-patient interaction [1]. Online medical consultation services comprise a novel channel through which physicians can offer more intense interactions to patients at lower costs [2,3]. On online health care platforms, physicians answer medical questions and share health care knowledge with patients in their spare time. Meanwhile, physicians can get both economic and social returns by offering their medical services, such as answering questions and sharing health care knowledge [4]. Therefore, if the patients live in remote areas far from a hospital or they need to go to the hospital, the online health care service can provide a more convenient and easy approach for patients to access health services. Online medical interactions, including both paid and free services, could promote increased respect and trust [5,6]. Physicians can build their personal reputation and receive recognition for providing free online medical consulting services [7]. This type of online prosocial behavior not only benefits physicians in a nonprofit way, such as through social returns, but also patients who express their health concerns online [2]. Therefore, online health care services extend traditional offline health services and satisfies the unfulfilled medical demands that offline health care fails to accomplish.

There are still many challenges in the development of the online health care industry. First, choosing an appropriate physician is critical for online patients [8]. However, because of information asymmetry and lack of professional health care knowledge, it is difficult for patients to ascertain a physician’s competency and service quality based on limited information and knowledge [9]. A physician’s online prosocial behavior could provide such information to help patients in the telemedicine market. Second, although physician online prosocial behavior is an indispensable resource for the development of telemedicine markets [4], participating in and contributing to the online medical marketplace is burdensome for physicians due to their heavy offline workloads. Thus, both patients and physicians encounter difficulties in participating in the online medical marketplace. Understanding how to enable physicians to make more online prosocial contributions has become a managerial agenda for telemedicine practitioners.

The introduction of monetary incentives may influence physicians’ prosocial behaviors through self-determination and image concerns [10]. Monetary incentives are increasingly adopted as a method of improving individual performance in many research domains [11-15]. Patients who are satisfied with a physician’s online service can pay a service fee to the physician. This monetary incentive can improve the reliability of service [6]. Moreover, this type of incentive can bring both reputational and monetary rewards for physicians, motivate their online contributions, and enhance their service quality. Therefore, introducing monetary incentives might have a positive effect on physician prosocial behavior in the telemedicine market.

Although physician online prosocial behavior has significance for online patients and society, there has been little research to explore it deeply. First, research on physician online prosocial behavior in telemedicine markets is scant. Previous research has been in a wide range of disciplines, such as economics [16] and marketing [17], but it has neglected the existence of the emerging telemedicine context. Second, although there has been extensive research exploring various factors for prosocial behavior—including situational factors, bystander effects [18], and individual factors such as cognitive capacities [19]—little research has investigated the impact of monetary incentives on physician online prosocial behavior. Furthermore, exploration on the effect of monetary incentives on physician behavior can give us a better understanding on the development of the online health care market. Physicians provide consultation services, knowledge, and information to help patients understand their diseases and obtain treatment, which can promote the development of telemedicine markets. Hence, it is important to investigate the role of monetary incentives on physicians’ online prosocial contributions. To fill these research gaps, the main research questions leading this study are can monetary incentives improve physicians’ prosocial behaviors in online medical consulting platforms and does this effect differ in the extent of physicians’ self-recognition and patients’ recognition?

## Methods

### Research Hypotheses

Our study investigated the impact of monetary incentives on physicians’ prosocial behaviors based on self-determination and image concerns theories [10].

Self-determination theory details intrinsic motivation and extrinsic motivation [10]. Intrinsic motivation means that one is motivated by one’s interest in an activity and inherent satisfaction, and extrinsic motivation refers to one’s behavior initiated and maintained by contingencies external to the person, such as tangible rewards and intangible rewards. Image concerns refer to an individual’s concerns with the perceptions of others. If individuals desire to be liked and respected by others, they would try to adjust their behaviors to signal good traits [12,16].

Generally, prosocial behavior is defined as a contributors’ actions that benefit other people or society [20]. Although evidence indicates that intrinsic motivation and extrinsic motivation have separate effects on prosocial behavior [15], they also interact with each other [21,22], especially when monetary incentives are introduced [11,23-25]. In particular, the motivation crowding-out theory shows that monetary incentives may have a negative effect on prosocial behavior by underlying intrinsic motivation [16]. Moreover, monetary incentives also induce extrinsic motivation and bring image concerns, according to previous studies [12,16,26]. In particular, if contributors receive extrinsic rewards for prosocial behaviors, they are suspected of acting prosocially primarily for financial reward rather than out of intrinsic motivation, such as pure altruism or concern for others’ well-being. The presence of extrinsic incentives spoils the presentation of a prosocial image and creates doubts regarding the contributors’ good deeds.

When making decisions, physicians would predict the outcomes of choosing different actions and may seek to draw lessons from consequences suffered both by themselves and others [27]. To avoid putting themselves in situations of image concern [28-30] or social pressure [31,32], they will care more about appearing prosocial to themselves. If individuals are looking to keep their good image and social approval, they would choose to engage in prosocial activities and contribute more prosocial behaviors after accepting extrinsic rewards. Usually, image concerns may be more dominant than crowding-out effects in social interaction settings.

In telemedicine markets, patients usually communicate with physicians to obtain medical advice and professional treatment, and physicians contribute free feedback to patients to promote their online presence and image [6,33]. Recently, a new type of service feedback has been applied to the telemedicine platforms—paid feedback. Patients may pay service fees to the physicians to encourage them to engage in online medical feedback. However, concentrating on paid feedback means that physicians’ online medical services may be just for monetary reward, thereby spoiling the signal of a prosocial image because free feedback indicates more about physicians’ prosocial tendencies. Therefore, after accepting monetary incentives, physicians would contribute more free feedback and increase their prosocial behaviors to display private preferences for others’ well-being and to avoid looking selfish and greedy. Thus, we hypothesized that the introduction of monetary incentives will improve physicians’ prosocial behaviors (hypothesis 1). However, the introduction of monetary incentives may not be equally important for all physicians and may differ by the extent of basic psychological needs.

Self-determination theory proposes that human beings have basic psychological needs for autonomy, competence, and relatedness, and that satisfaction of these basic psychological needs provides the nutriments for intrinsic motivation and internalization of extrinsic motivation [10]. Therefore, work climates that support the satisfaction of these needs will promote a person’s enjoyment of activities (intrinsic motivation) and the autonomous self-regulation of behaviors (internalization of extrinsic motivation) [34]. Human behaviors can be characterized and determined in terms of the degree of intrinsic motivation and extrinsic motivation [35]. Intrinsic motivation will facilitate good work outcomes, such as effective performance and positive work-related attitudes. Gagné [36] showed that satisfaction of needs will orient people toward paying more attention to others, thus making them more likely to engage in prosocial behaviors.

**Figure 1:**
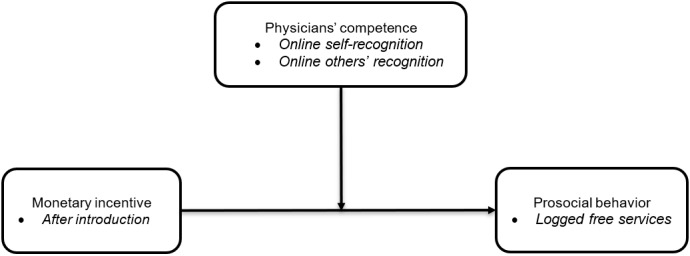
Research framework of the impact of introducing monetary incentives on physician online prosocial behavior.

Toubia and Stephen [30] suggest that competence should encompass the sense of self-worth and social acceptance based on a user’s activities on online platforms. In other words, competence is evaluated by oneself and others. Therefore, satisfaction of competence is based on self-recognition and others’ recognition of a user’s ability and performance. In telemedicine markets, physicians offer online medical consulting services to help patients understand their diseases and get treatment. The competence satisfaction of physicians is determined by their feelings of competence to master online feedback and provide professional treatment, and patient recognition of their past work performance [9]. According to self-determination theory, physicians’ prosocial behavior may vary with the level of satisfaction of basic psychological needs (eg, competence satisfaction). Physicians have higher satisfaction in their competence if they obtain higher self-recognition and recognition from others of their competence. Therefore, they are more likely to pay attention to others, thereby contributing free feedback and behaving prosocially. Based on this, we hypothesized the following: the extent of online self-recognition of physicians’ competence strengthens the effect of monetary incentives on their prosocial behaviors (hypothesis 2) and the extent of online others’ recognition of physicians’ competence strengthens the effect of monetary incentives on their prosocial behaviors (hypothesis 3). Our research framework is shown in Figure 1.

### Research Design

The variance in the timing of monetary incentive appearance across physicians provides a unique quasi-experimental opportunity to estimate its influence on physician’s online prosocial behavior. With the entry of monetary incentives in a particular month as the treatment, physicians who had at least one entry were the treatment group (ie, physicians with incentive), and those without any entry were the control group (ie, physicians without incentive). We used a difference-in-differences (DID) approach to represent the quasi-experiment [37]. In our DID design, the first difference was between treated physicians and control physicians, and the second difference was between the periods before and after incentive. The double differencing eliminated the potential biases that may come from inherent trends in the prosocial behaviors of physicians.

### Data and Variables

**Figure 2:**
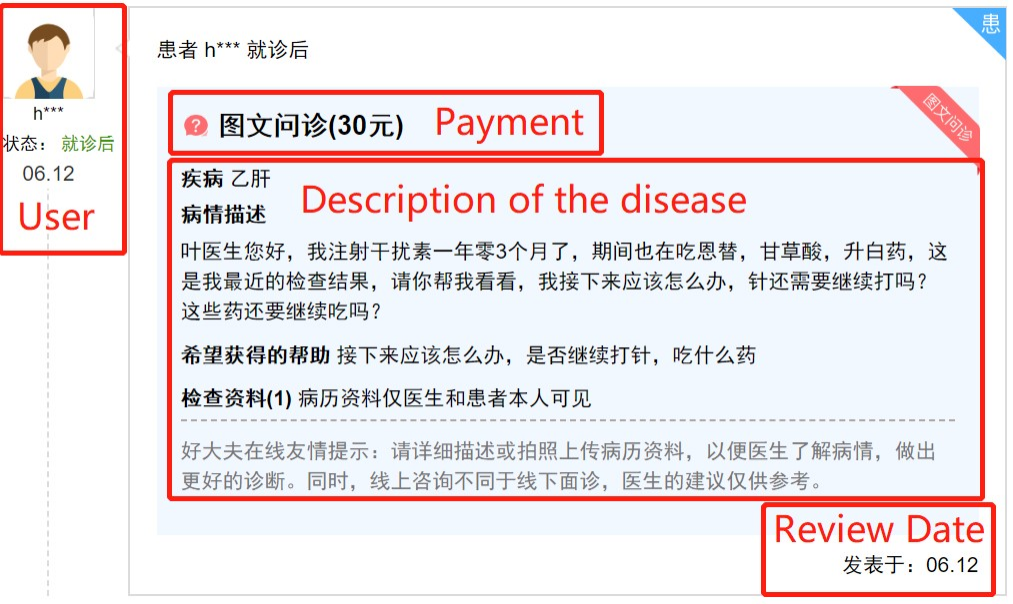
Description of the paid consultation services.

This study collected data from a leading online health care platform called Haodf in China. The functions of the platform include online medical consultation, appointment referral, medical information inquiries, knowledge sharing of medical science, physician recommendations, and so forth. In addition, this platform offers a unique institutional setting to separate free and paid consultations. We identified a paid consultation service as a significant sign of payment as shown in Figure 2. In a free consultation, patients received slow and limited responses from physicians; in a paid consultation, patients communicated immediately with physicians. We selected 26,543 physicians in 3851 hospitals who had provided paid consultation services as the target sample. We developed a crawler to collect the historical data of physicians from November 2006 to December 2017 (133 months) and their attributed data on January 2018 from Haodf. The definitions and statistical descriptions of major variables are shown in Table 1. Additionally, Table 2 presents the correlations of the main variables in the research model, which indicates that there was no significant multicollinearity among the independent variables.

#### Dependent Variables and Independent Variables

Physicians’ prosocial behaviors were the free consulting services offered by physicians in an online health care community, measured by the logged volume of free answers in a given month as the dependent variable. The introduction of online monetary incentives was the key independent variable of interest in our estimation.

#### Moderators and Control Variables

To explore the heterogeneous effects of monetary incentives on physicians’ prosocial behavior, we introduced two streams of moderators, including high price and high rated. High price indicated the extent of physicians’ self-recognition measured by the pricing strategy of consulting established by physicians. High rated was the extent of others’ recognition of a physician measured by the online rating posted by patients. Several control variables were considered to ensure the model robustness; examples include patient votes, letters of thanks, affiliated hospital level, and professional title.

### Research Model

For a physician *i* in month *t,* we modeled the entry effect of monetary incentives as follows:

Y_it_ = β(monetary incentive)_it_ + γ(monetary incentive)_it_ × Z_it_ + μ_i_ + v_t_ + ε_it_

where *Y* is the logarithm of monthly free consulting services (prosocial behavior). The monetary incentive dummy variable indicates whether physician *i* has experienced at least one monetary incentive. *Z* is a vector of moderators, including high price and high rated. We account for the unobserved heterogeneity across physician and temporal trends that may be correlated with both monetary incentives and the prosocial behaviors of physicians; *μ* represents the physician-level fixed effects to account for time-invariant characteristics of physicians, *v* is a vector consisting of both month trends and year-month fixed effects to control for temporal trends or shocks that apply to the online medical market, and *ε* is an idiosyncratic error term. The treatment effect and moderating effects are identified by the coefficient β and the vector of coefficients *γ,* respectively. We clustered robust standard errors at the physician level to account for the potential correlation in the standard errors within physicians [38,39].

**Table 1:** Definitions and summary statistics of variables (N=777,110).

Variable		Definition	Mean (SD)	Range
**Dependent variable**				
	Prosocial behavior	Logged number of free services offered by a physician in a given month	1.600 (1.958)	0-10.788
**Independent variable**				
	Monetary incentive	Dummy variable indicating whether a physician started receiving actual monetary income in a given month	0.307 (0.461)	0-1.000
**Moderators**				
	High price^a^	Dummy variable indicating whether the consulting price is high for a physician in online medical consulting platform	0.279 (0.449)	0-1
	High rated^a^	Dummy variable indicating whether a physician is high rated by the users in online medical consulting platform	0.451 (0.498)	0-1
**Control variables**				
	Patient votes	Logged number of votes showing praise given by patients to a physician	2.815 (1.479)	0-7.720
	Letters of thanks	Logged number of letters of thanks that a physician received in a given month	0.162 (0.471)	0-5.273
	High hospital level^a^	Dummy variable indicating whether a hospital is designated by the Chinese government as a “third-level grade-A” level	0.765 (0.424)	0-1
	Professional title	Official clinic title certified by the national agency with uniform standards; Four stages exist for clinic titles: archiater (4), associate archiater (3), chief physician (2), resident physician (1), and none (0).	3.073 (0.886)	1-4

^a^High price, high rated, and high hospital level are split by their mean values.

**Table 2:** Statistical analysis of pairwise correlation of variables.

Variable	1	2	3	4	5	6	7	8
1. Prosocial behavior	—							
2. Monetary incentive	.461^a^	—						
3. Votes	.407^a^	.215^a^	—					
4. Letters	.497^a^	.34^a^	.353^a^	—				
5. High price	.195^a^	.106^a^	.446^a^	.175^a^	—			
6. Title	.035^a^	−.048^a^	.371^a^	.022^a^	.229^a^	—		
7. High rated	.214^a^	.127^a^	.603^a^	.222^a^	.350^a^	.246^a^	—	
8. Hospital level	.010^a^	.016^a^	.168^a^	.037^a^	.108^a^	.100^a^	.207^a^	—

^a^*P*<.01.

## Results

### Model-Free Evidence

**Figure 3:**
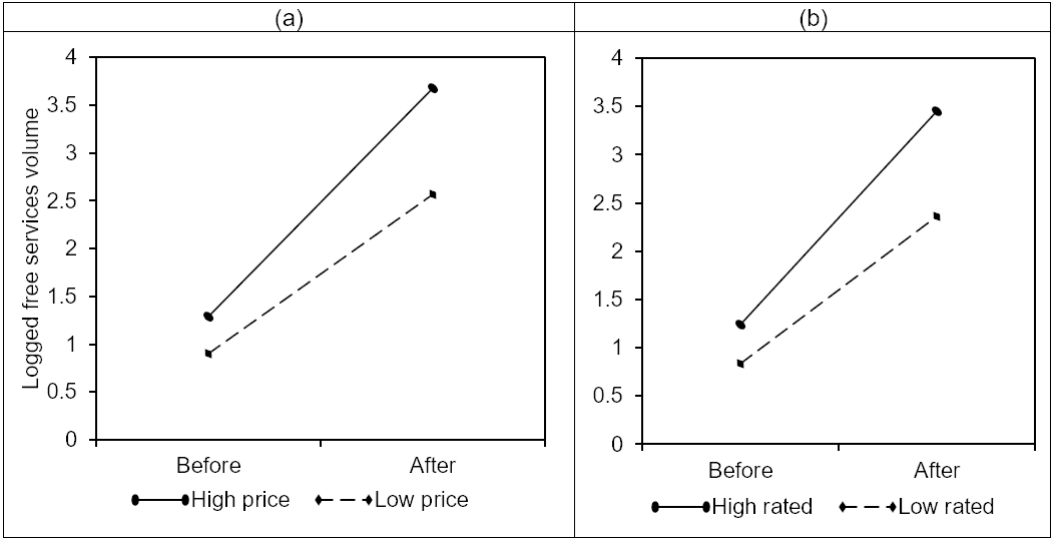
Model-free comparison of free services volume for physicians by (a) price setting and (b) rating.

We compared the intensity of physicians’ online prosocial behaviors before and after the introduction of monetary incentives. To illustrate the moderation effects of high price and high rated, we generated a set of plots using the physicians in our data who had experienced monetary incentives (ie, treatment physicians). Figure 3 shows changes in the physicians’ online prosocial behavior after the introduction of monetary incentives by the level of physicians’ pricing strategy and online rating. The y-axis is the logged volume of free services offered by physicians. The amount of online prosocial behavior for both high self-recognition (high price) and low self-recognition (low price) physicians increased after the introduction of monetary incentives; the increase for high self-recognition physicians was much higher than for low self-recognition physicians. Similarly, the online prosocial behavior volume increased more for high-rated physicians than low-rated physicians. These patterns are consistent with the monetary incentive effects predicted in our three hypotheses and provide preliminary support to these hypotheses.

### Model Estimation

We report the estimated effects of monetary incentives on physicians’ online prosocial behavior in Table 3. First, we employed the ordinary least squares and the fixed-effects specifications to estimate the main effect of monetary incentives. Both specifications showed consistent results: the introduction of monetary incentives led to a significant increase in physicians’ online prosocial behaviors, supporting hypothesis 1. Monetary incentives increased physicians’ concerns about their prosocial self-image; they wanted to make more prosocial contributions to strengthen their online images. If the online image-strengthening effect held, there should be a stronger effect of introductory monetary incentives for physicians with high levels of self-recognition and others’ recognition. Estimates with self-recognition and others recognition confirm our conjecture; therefore, hypotheses 2 and 3 are supported. The online image-strengthening effect provided by introductory monetary incentives worked better, especially for physicians with high image demands. The combined model also affirmed our hypotheses.

### Robustness Check

Although we controlled a set of observable attributes along with the physicians’ and time-fixed effects, a potential problem with our DID design was that physicians are different. A particular concern was that physicians who enrolled later may have been attracted by the introductory incentive policy, or there was a different trend that caused a selection bias in our estimation.

To check the robustness of our research model, we used matching methods to select similar physicians from our control and treatment groups to replicate the main analyses shown in Table 4 [40,41]. Specifically, we used the physicians’ personal characteristics (ie, seniority, hospital ranking, and registration date) and contribution characteristics (ie, gifts and letters of thanks received, total patients replied) to match the physicians. Finally, there were 5131 physicians matched by a caliper match (a caliper of 0.001). Table 4 presents the estimated results of the robustness check, which confirmed our findings of monetary incentives on physicians’ online prosocial behavior. Therefore, the results of the robustness check are consistent with the results of the main model.

**Table 3:** Estimation results of the impact of monetary incentives on physicians’ online prosocial behavior (N=777,110).^a^

Variable	Prosocial behavior				
	Ordinary least squares	Fixed effects	Self-recognition	Recognition from others	Combined model
Monetary incentive	1.498^b^ (0.019)	1.057^b^ (0.019)	0.959^b^ (0.022)	0.876^b^ (0.026)	0.842^b^ (0.026)
Monetary incentive × high price	—^c^	—	0.275^b^ (0.037)	—	0.188^b^ (0.040)
Monetary incentive × high rated	—	—	—	0.325^b^ (0.034)	0.267^b^ (0.036)
Control variables					
Letters	1.259^b^ (0.011)	1.167^b^ (0.011)	1.152^b^ (0.011)	1.143^b^ (0.011)	1.137^b^ (0.011)
Votes	0.345^b^ (0.008)	—	—	—	—
High price	0.071^b^ (0.023)	—	—	—	—
Title	−0.129^b^ (0.009)	—	—	—	—
High rated	−0.167^b^ (0.021)	—	—	—	—
High hospital level	−0.190^b^ (0.019)	—	—	—	—
Physician fixed effects	No	Yes	Yes	Yes	Yes
Month trends	Yes	Yes	Yes	Yes	Yes
Month fixed effects	Yes	Yes	Yes	Yes	Yes
*R*^2^	.413	.230	.231	.232	.232

^a^Robust standard errors are in parentheses.

^b^*P*<.01.

^c^Not applicable.

**Table 4:** Estimation results of robustness check using matched samples (N=425,469).^a^

Variable	Prosocial behavior				
	Ordinary least squares	Fixed effects	Self-recognition	Recognition from others	Combined model
Monetary incentive	1.622^b^ (0.032)	0.989^b^ (0.029)	0.853^b^ (0.034)	0.767^b^ (0.042)	0.709^b^ (0.042)
Monetary incentive × high price	—^c^	—	0.319^b^ (0.051)	—	0.242^b^ (0.054)
Monetary incentive × high rated	—	—	—	0.349^b^ (0.050)	0.279^b^ (0.053)
Control variables	—	—	—	—	—
Letters	1.186^b^ (0.016)	1.157^b^ (0.018)	1.132^b^ (0.018)	1.125^b^ (0.018)	1.112^b^ (0.018)
Votes	0.401^b^ (0.013)	—	—	—	—
High price	0.041 (0.030)	—	—	—	—
Title	−0.018 (0.021)	—	—	—	—
High rated	−0.192^b^ (0.029)	—	—	—	—
High hospital level	−0.101^b^ (0.030)	—	—	—	—
Physician fixed effects	No	Yes	Yes	Yes	Yes
Month trends	Yes	Yes	Yes	Yes	Yes
Month fixed effects	Yes	Yes	Yes	Yes	Yes
*R*^2^	.403	.223	.225	.225	.226

^a^Robust standard errors are in parentheses.

^b^*P*<.01.

^c^Not applicable.

## Discussion

### Summary of Findings

This study investigated the influence of monetary incentives on physicians’ online prosocial behaviors. Based on self-determination theory, we developed three research hypotheses and established an empirical model based on a DID design. The results of our research model support our hypotheses. Accordingly, this work provides three key findings. First, we found that the introduction of monetary incentives has a positive effect on physicians’ online prosocial behavior (as measured by free services offered). Second, this promotion effect is enhanced by physicians’ self-recognition of their personal medical competence (as measured by higher price setting). Third, the extent of patients’ recognition of physicians’ medical competence (as measured by higher rating) also can strengthen the positive effect of introductory monetary incentives on physicians’ online prosocial behaviors.

### Discussion of Research Results

Prosocial behavior refers to any behavior that is beneficial to others and society. Prior studies mainly focus on contribution to charity [12,21], medical treatment in hospital [20], endowment of money [42], volunteerism for the American Red Cross [26], and blood donations [13]. Recently, some researchers investigated a broader range of prosocial behavior types, such as individuals’ knowledge-sharing behavior [43] and content contribution in social media [14,30]. In telemedicine markets, physicians provide free online medical consulting services, which are a type of prosocial behavior through the internet. However, little research has investigated physicians’ prosocial behaviors in telemedicine markets. Our study addressed this gap based on self-determination theory and found a new factor (ie, introduction of monetary incentives) that significantly affects physicians’ online prosocial behaviors.

Moreover, monetary incentives are often used to encourage contributors to improve their prosocial behaviors [13]. It is one of the key external incentives of prosocial performance. However, several studies have found that monetary incentives may backfire [42,44], and some researchers argue that rewards will introduce image concerns about appearing “greedy” instead of “prosocial” [12,13,16,26]. However, the effect of monetary incentives on prosocial behavior is a joint function of internal psychological processes and environmental factors. In telemedicine markets, online medical feedback is a repeatable behavior, meaning that physicians can contribute more free feedback to maintain and compensate for their prosocial images after accepting monetary rewards. Thus, we find that monetary incentives have a positive effect on the intensity of physicians’ online prosocial behaviors, which provides new evidence against the backfire of monetary incentives in an online health care context.

In addition, the introduction of monetary incentives may not be equally important for all physicians, and their differences should be taken into consideration. According to self-determination theory, the satisfaction of competence makes them more likely to engage in prosocial behaviors. As competence is evaluated by oneself and others, satisfaction of competence is based on self-recognition and others’ recognition of a user’s ability and performance. In particular, if physicians obtain higher self-recognition and recognition from others of their competence, they will have higher satisfaction of competence. Therefore, they will be more likely to contribute free feedback and behave prosocially. Based on the previous discussion, we found that the extent of self-recognition and patients’ recognition on physicians’ competence can strengthen the positive effect of introductory monetary incentives on physicians’ online prosocial behaviors, which gives us a better understanding of the mechanisms behind these behaviors.

### Limitations and Future Research

There are several limitations of this study. First, our observations are of only Chinese physicians. The incentive effects may differ in other countries due to cultural differences. Future studies can investigate this issue by leveraging cross-platform datasets. Second, this preliminary study investigates the general effects of incentives on physicians’ online prosocial behaviors. More detailed settings, such as comparing online and offline environments, should be applied in future studies.

### Contributions

This study contributes to the literature in several ways. First, to our knowledge, this is the first study to investigate physicians’ prosocial behaviors in the telemedicine context, which adds to both streams of eHealth and prosocial behavior. Although abundant studies have examined prosocial behaviors in offline markets [12,13,26], few have explored it in online markets, especially in an online health care context. In filling this research gap, our research extends the current understanding of online prosocial behavior through the consideration of free online medical consulting services offered by physicians. Second, we enrich the existing literature of factors that affect online prosocial behavior [26,30,32]. This research examined whether and how the introduction of a monetary incentive affects the online prosocial performance of physicians, which extends the current studies on online prosocial performance and related influence factors. The results confirm the effects of introductory monetary incentives on physicians’ online prosocial performances and encourage future studies to consider it as an important perspective when studying online prosocial behavior. Third, this study deepens the literature of online prosocial behavior in specific mechanisms by considering the extent of recognition on physicians’ competence [2,43]. We found that the promotion effect of monetary incentives on physicians’ online prosocial behaviors is enhanced by physicians’ self-recognition, and patients’ recognition of physicians’ medical competence. This study illuminates that offering online prosocial behaviors is an effective way to present real quality information and build reputation for physicians, which is an important insight in the existing literature of both marketing and eHealth. This provides novel insights into the future studies that tend to take specific business processes into account when studying online health care.

This study also offers some practical implications. First, this study indicates an effective approach to increase physicians’ online prosocial behaviors by introducing monetary incentives. This can prompt physicians to improve their allocations of service. Ultimately, patients benefit more from these extra online prosocial behaviors. Second, our findings will shed light on the facilitating roles of physician traits by testing several practice-oriented variables (price setting and rating value), which provide valuable implications to practitioners. Physicians of different types can take corresponding measures to promote themselves by providing prosocial behaviors in online platforms. Due to the imbalance issue of increasing online medical demands and limited eHealth system resources globally, physicians’ online prosocial behaviors are effective ways to compensate for medical services online and offline.

### Conclusions

Our study investigates physicians’ online prosocial behaviors through self-determination theory embedded in an online health care platform. We extend self-determination theory in the online health care context and demonstrate the relationship between incentive mechanisms and the prosocial behaviors of physicians. The preliminary results support our theory-based model. We found that the introduction of monetary incentives has a positive effect on the volume of physicians’ online prosocial behaviors, and the extent of self-recognition and others’ recognition of physicians’ competence can strengthen this promotion effect. This means that physicians with high self-recognition and others’ recognition will make more prosocial contributions in online health care platforms.

## Abbreviations

DID: difference in differences
